# Supplementary material for: Complex‐centric proteome profiling by SEC‐SWATH‐MS
Source: Mol Syst Biol. 2019 Jan 14;15(1):e8438. doi: 10.15252/msb.20188438 (PMC6346213; doi:10.15252/msb.20188438)
Supplement: Supplementary file 8 — Dataset EV7 [file MSB-15-e8438-s008.zip › feature_plots_string/P78537.pdf]

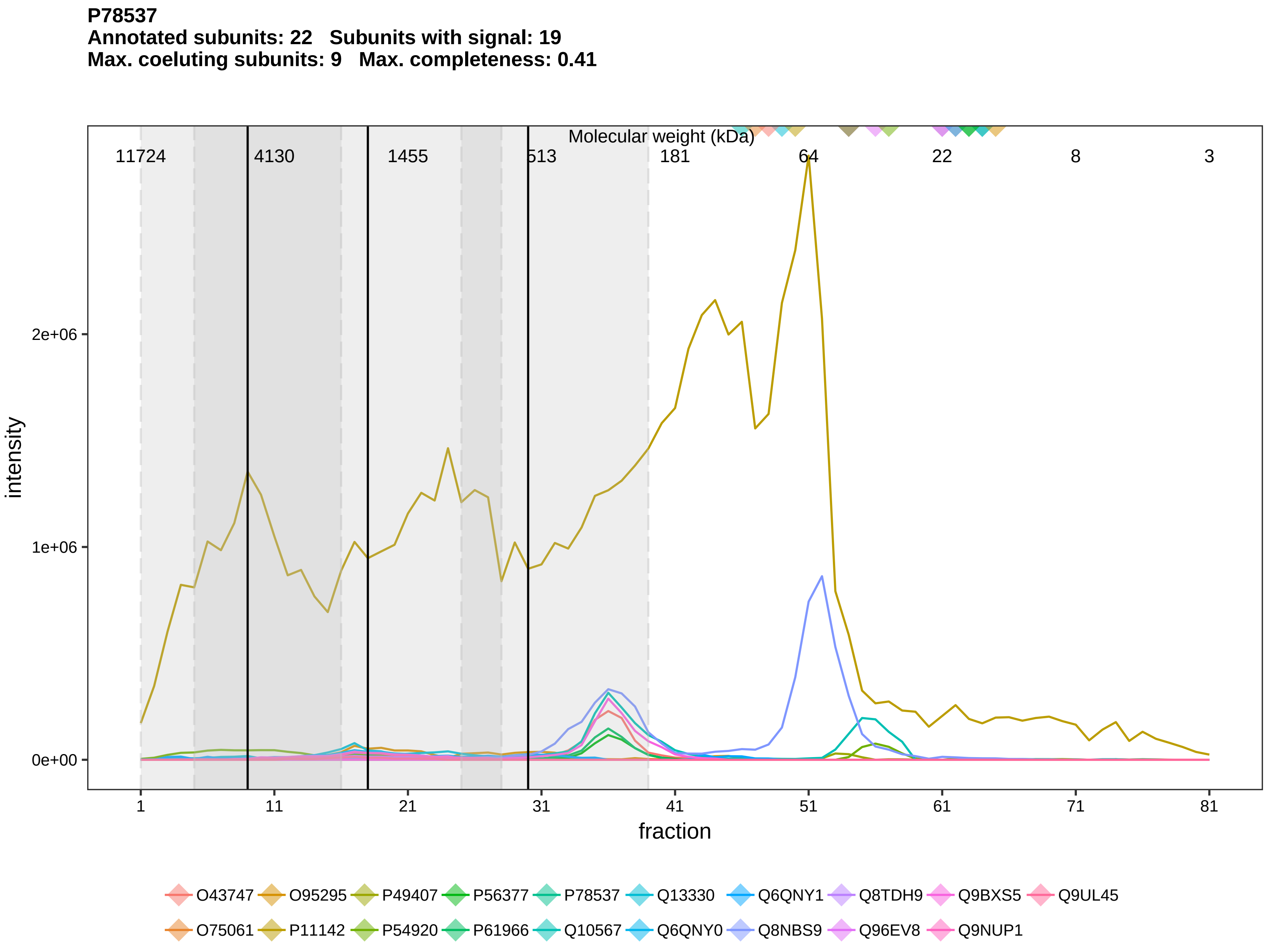

P78537
Annotated subunits: 22 Subunits with signal: 19
Max. coeluting subunits: 9 Max. completeness: 0.41
Molecular weight (kDa)
11724
4130
1455
513
181
64
22
8
3
2e+06
1e+06
0e+00
intensity
1
11
21
31
41
51
61
71
81
fraction
O43747
O95295
P49407
P56377
P78537
Q13330
Q6QNY1
Q8TDH9
Q9BXS5
Q9UL45
O75061
P11142
P54920
P61966
Q10567
Q6QNY0
Q8NBS9
Q96EV8
Q9NUP1
